# Urinary gonadotrophins: a useful non-invasive marker of activation of the hypothalamic pituitary-gonadal axis

**DOI:** 10.1186/1687-9856-2012-10

**Published:** 2012-05-04

**Authors:** Jane D McNeilly, Avril Mason, Sheila Khanna, Peter J Galloway, S Faisal Ahmed

**Affiliations:** 1 Department of Biochemistry, Royal Hospital for Sick Children, Glasgow, UK; 2 Developmental Endocrinology Research Group, Royal Hospital for Sick Children, Glasgow, UK; 3 Section of Child Health, University of Glasgow, Royal Hospital for Sick Children, Glasgow, G3 8SJ, UK

**Keywords:** Adolescence, Assessment, FSH, LH, Puberty

## Abstract

**Background:**

Non-invasive screening investigations are rarely used for assessing the activation and progression of the hypothalamic-pituitary gonadal axis through puberty. This study aimed to establish a normal range for urinary gonadotrophins in children progressing through puberty.

**Methods:**

Urine samples were collected from 161 healthy school children (76 boys, 85 girls) aged 4–19 yrs. Height and weight were converted to standard deviation score. Pubertal status, classified by Tanner staging, was determined by self-assessment. Urinary gonadotrophins were measured by chemiluminescent microparticle immunoassay. Results were grouped according to pubertal status (pre-pubertal or pubertal).

**Results:**

Of the 161 children, 50 were pre-pubertal (28 boys; 22 girls) and 111 were pubertal (48 boys; 63 girls). Overall, urinary gonadotrophins concentrations increased with pubertal maturation. All pre-pubertal children had a low urinary LH:Creatinine ratio. LH:Creatinine ratios were significantly higher in pubertal compared to pre-pubertal boys (*p*<0.001). In girls, FSH:Creatinine ratios were significantly higher in the pubertal group (*p* = 0.006). However, LH:FSH ratios were a more consistent discriminant between pre-pubertal and pubertal states in both sexes (Boys 0.45 pubertal vs 0.1 pre-pubertal; girls 0.23 pubertal vs 0.06 pre-pubertal).

**Conclusion:**

Urinary gonadotrophins analyses could be used as non-invasive integrated measurement of pubertal status which reflects clinical/physical status.

## Introduction

At present there are no practical non-invasive screening investigations to assess the activation and progression of the hypothalamic-pituitary-gonadal (HPG) axis. Routine investigations rely on the measurement of serum gonadotrophins before and after stimulation of gonadotrophic secretion by GnRH agonists and analysis of various different plasma steroids. These tests are expensive, invasive and often require a visit to the hospital. In addition, they are not suited for situations where there is a need for serial biochemical monitoring.

Analysis of urinary gonadotrophins in children was first reported almost 50 years ago [1,2] when highly labour-intensive methods, requiring numerous pre-treatment procedures and 24 hr urine collections were necessary. Subsequently, the development of more sensitive and specific automated immunoassays for gonadotrophins has led to a movement away from urine to serum as the sample of choice. However, measurement of urinary gonadotrophins may be of benefit in particular situations such as longitudinal studies [3,4], neonates [5] and pubertal assessment [6-10]. The aims of this study were, initially, to determine whether measurement of urinary gonadotrophins could differentiate between physically pre and post-pubertal children and, secondly, to establish and validate normal urinary gonadotrophin ranges for healthy pre and post-pubertal children.

## Methods

### Study description

A total of 161 healthy children, 76 boys and 85 girls, aged between 4 and 19 years were recruited from schools in different areas of Glasgow, UK, as part of a study examining bone health over a 1 year period (2005–6). The study was approved by the local research ethics committee and each child and their parents gave informed assent and consent respectively. Height and weight were recorded at enrolment and converted into standard deviation score (SDS) using 1990 British children standards [11]. Pubertal status was determined by children and/or parents using self-assessment charts and classified according to Tanner staging as pre-pubertal (Tanner stage 1); early pubertal (Tanner stage 2–3); late pubertal (Tanner stage 4–5). A single non-timed urine specimen was collected at enrolment and stored at −20°C for future analyses.

### Methodology

Urinary LH and FSH were measured using a chemiluminescent microparticle immunoassay (CMIA) from Abbott on the Architect ci8200 (Abbott Laboratories Diagnostics, IL, USA). The lower limit of detection for FSH and LH was 0.1 IU/L and inter and intra-assay precision (CV) for both LH and FSH were less than 5% at levels of 0.2 and 2.3 IU/L for LH and 0.6 and 4.7 IU/L for FSH. Analytical sensitivity of the assays was 0.07 IU/L and 0.05 IU/L for LH and FSH respectively. Urinary creatinine was measured using the modified Jaffe method with a lower limit quantitation of 0.35 mmol/L. Intra and inter-assay CVs were 2.7% and 3.5% respectively (5.8 and 12.5 mmol/L). Samples with a creatinine <1.0 mmol/L were regarded as too dilute for analysis. FSH and LH results were corrected for creatinine to take into account urine concentration.

### Statistical analysis

All data were expressed as median (10^th^ and 90^th^ centiles) and were analysed using SPSSv10.0 (SPSS Inc., Chicago, IL, USA). Statistical analyses were performed with the nonparametric Mann–Whitney *U* test for between-group comparisons for continuous variables. A p value of less than 0.05 was considered statistically significant.

## Results

### General characteristics

The demographics of the cohort according to pubertal staging are shown in Table 1. Due to the small numbers in the early and late pubertal groups, children were classified as being pre-pubertal (Tanner stage I) or pubertal (Tanner stages II-V). Of the 161 children, 50 were pre-pubertal (28 boys) with median ages of 7.8 yrs (10^th^,90^th^ centiles; 6.2, 11.6) and 8.4 yrs (5.5, 12.6.) in girls, respectively. Of the remaining 111 pubertal children (48 boys), the median age for boys was 15.0 yrs (12.2, 17.4) and greater than that for girls (median, 13.6 yrs; 10.7, 17.0). The median height and BMI SDS data as shown in Table 1, illustrate that our cohort is representative of a general childhood population. Median height SDS was similar in both pre-pubertal boys and girls (boys −0.1 vs girls −0.3), however the range was wider for boys (−1.2, 1.3 than girls (−1.5, 0.8). In pubertal children, median height SDS was higher in boys than girls (boys 0.3 (−0.9, 1.2) vs girls −0.1 (−0.9, 1.4). Likewise, median BMI SDS were similar in pre-pubertal children (boys 0.4 (−1.1, 1.9) vs girls 0.2 (−1.8, 1.4), with pubertal boys having a slightly higher median BMI than girls (boys 0.6 (−0.8, 1.5) vs girls 0.1 (−0.8, 1.6).

**Table 1:** Patient demographics grouped by pubertal status

	**Pre-pubertal** **boys**	**Pre-pubertal** **girls**	**Pubertal** **boys**	**Pubertal** **girls**
**N**	**28**	**22**	**48**	**63**
**Age (yrs)** (Median, 10^th^ & 90^th^ centiles)	7.8 (6.2, 11.6)	8.4 (5.5, 12.6)	15.0 (12.2, 17.4)	13.6 (10.7, 17.0)
**Height SDS** (Median, 10^th^ & 90^th^ centiles)	− 0.1 (− 1.2, 1.3)	−0.3 (−1.5, 0.8)	0.3 (−0.9, 1.2)	−0.1 (−0.9, 1.4)
**BMI SDS** (Median, 10^th^ & 90^th^ centiles)	0.4 (− 1.1, 1.9)	0.2 (− 1.8, 1.4)	0.6 (− 0.8, 1.5)	0.1 (− 0.8, 1.6)
**Pubertal Stage** II/III/IV/V			10/11/19/8	12/20/19/12

Data reported as median (10^th^ and 90^th^ centiles). Height and BMI SDS score are based on the British 1990 growth reference data [11].

### Urinary gonadotrophins in relation to pubertal status

**Figure 1:**
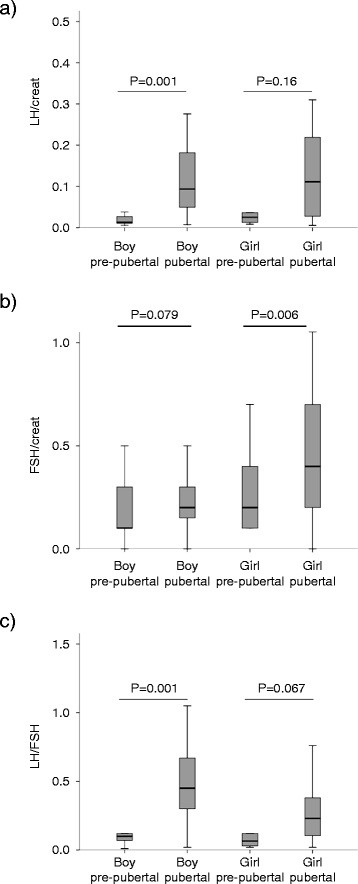
**a-c - Urinary gonadotrophins grouped according to pubertal status.** a) Median LH:Creatinine, b) FSH:Creatinine and c) LH:FSH ratios grouped according to pubertal status. The box and whisker plots represent the median (heavy line), interquartile (box) and range (whiskers).

Of the original 161 samples, 113 (70%) had measurable LH and FSH. LH was undetectable (<0.1 IU/L) in 46 samples (20 boys; 26 girls) with only 2 samples (girls) having undetectable FSH (<0.1 IU/L). None of the samples were regarded too dilute for analysis (creatinine < 1.0 mmol/L). Overall, the median levels of urinary gonadotrophins increased with pubertal maturation. All pre-pubertal children had very low LH:Cr ratio with little spread (median LH:Cr; boys 0.01 (centiles 0.0, 0.10), girls 0.03 (0.01, 0.85) (Figure 1a). A significant difference between pre-pubertal and pubertal LH:Cr ratio was observed in boys with those in puberty having higher LH concentrations (median LH:Cr; pre-pubertal 0.01 (0.0, 0.10), pubertal 0.09 (0.02, 0.24) *p*< 0.001; Figure 1a). A similar pattern was seen in girls but the difference was not significant (median LH:Cr; pre-pubertal 0.03 (0.01, 0.85), pubertal 0.11 (0.11, 0.52) *p*< 0.16; Figure 1a). Urinary FSH:Cr ratios showed a similar pattern with ratios being higher in children undergoing puberty (Figure 1b). The FSH:Cr ratio was higher in pubertal than pre-pubertal girls (median FSH:Cr; pre-pubertal 0.19 (0.10, 0.65) vs pubertal 0.45 (0.14, 1.19); *p* =  0.006 Figure 1b). There was, however, no significant difference in FSH:Cr ratio between the pre-pubertal and pubertal boys (*p* = 0.079). Urinary LH:FSH ratio clearly differentiated between physically pre-pubertal and pubertal children (Figure 1c). The LH:FSH ratio was higher in the pubertal than in pre-pubertal group in both sexes, reaching significance in boys (median LH:FSH pre-pubertal 0.1 (0.05, 0.37) vs pubertal 0.45 (0.14, 1.00); *p* = 0.001) but not girls (median LH:FSH pre-pubertal 0.06 (0.02, 0.61) vs pubertal 0.23 (0.05, 0.53); *p* = 0.067).

## Discussion

This study was designed to determine whether measurement of urinary gonadotrophins could distinguish between physically pre-pubertal and pubertal healthy children. Our data demonstrated that urinary gonadotrophins could clearly differentiate between pre-pubertal and pubertal status with ratio of LH:FSH proving to be a better discriminator than either LH or FSH:creatinine.

Importantly, our results are similar to those previously published in peri-pubertal children despite using non-extracted, random urines samples and performing the analyses on a routine automated immunoassay [3,6,8,9]. A previous report by Kujper et al. investigating neonatal gonadotrophins showed a strong correlation between urinary and serum FSH and LH measurements using the same Abbott Architect methods used in our study, confirming the suitability of this assay for urinary analysis [5].

Urinary LH excretion showed marked difference between pre-pubertal and pubertal children with pre-pubertal children having consistently low LH concentrations. Of our cohort, 46 children had undetectable LH (<0.1 IU/L). LH:Creatinine ratio was significantly higher in pubertal than pre-pubertal boys but not girls, despite median LH:Creatinine levels being similar (Figure 1a). The lack of significance between pre-pubertal and pubertal girls is likely due to the episodic gonadotrophin excretion pattern that is unique to early and mid-puberty [9]. Due to the random nature of the urine collection we may have missed these peaks in some children. Ideally first-void morning urine samples would have provided a better reflector of hormonal excretion over the preceding night.

In contrast to LH, urinary FSH excretion was more variable in all groups with no significant difference in excretion between pre-pubertal and pubertal boys. Both pre-pubertal and pubertal girls had higher FSH excretion than their male counterparts, with FSH:Creatinine differing significantly between pre-pubertal and pubertal girls. These results are consistent with previous studies which reported in girls that FSH excretion rose with significant fluctuations with the onset of puberty with levels remaining greater than LH until peri-menarche [3,8,9].

In our study, the LH:FSH ratio proved the best discriminator between physically pre-pubertal and pubertal children with pubertal children having higher ratios than pre-pubertal children. The advantage of this ratio over LH or FSH:Creatinine alone is likely due to the consistently low pre-pubertal urinary LH excretion in both genders. A possible reason why the difference between pre-pubertal and pubertal LH:FSH ratio in girls just failed to reach significance is the wider range of FSH:Creatinine ratios in the girls compared to the boys.

It is important to note that some pre-pubertal children had biochemistry suggestive of hypothalamic-pituitary-gonadal activation confirming previous observations that hormonal changes associated with puberty precede external physical clinical signs of puberty [6,9,12].

## Conclusions

Despite not being routinely utilised as a screening investigation for HPG activation, this study has demonstrated that urinary gonadotrophin analysis can be used as a non-invasive integrated measurement of pubertal status which reflects clinical/physical status. Furthermore, using urine samples rather than plasma overcomes the issues of sample insufficiency which is a particular problem in neonates. Future potential applications for urinary gonadodotrophin analysis include monitoring efficacy of treatment in precocious puberty and longitudinal studies of pubertal development. However further studies are required to determine the clinical usefulness in these patient cohorts.

## Abbreviations

LH = Luteinizing hormone; FSH = Follicle stimulating hormone; Cr = Creatinine.

## Competing interests

The authors declare they have no competing interests.

## Authors' contributions

JM performed the analysis and wrote manuscript. AM participated in the design of the study, performed the statistical analysis and helped to draft the manuscript. SFA conceived of the study, participated in its design and coordination and helped to draft the manuscript. SK collected samples. All authors read and approved the manuscript.
